# γ‐Secretase Dependent Nuclear Targeting of Dystroglycan

**DOI:** 10.1002/jcb.25537

**Published:** 2016-03-31

**Authors:** Daniel Leocadio, Andrew Mitchell, Steve J. Winder

**Affiliations:** ^1^ Department of Biomedical ScienceUniversity of SheffieldWestern BankSheffield S10 2TNUnited Kingdom

**Keywords:** DYSTROGLYCAN, PROTEOLYSIS, PROSTATE CANCER

## Abstract

Dystroglycan is frequently lost in adenocarcinoma. α‐dystroglycan is known to become hypoglycosylated due to transcriptional silencing of LARGE, whereas β‐dystroglycan is proteolytically cleaved and degraded. The mechanism and proteases involved in the cleavage events affecting β‐dystroglycan are poorly understood. Using LNCaP prostate cancer cells as a model system, we have investigated proteases and tyrosine phosphorylation affecting β‐dystroglycan proteolysis and nuclear targeting. Cell density or phorbol ester treatment increases dystroglycan proteolysis, whereas furin or γ‐secretase inhibitors decreased dystroglycan proteolysis. Using resveratrol treatment of LNCaP cells cultured at low cell density in order to up‐regulate notch and activate proteolysis, we identified significant increases in the levels of a 26 kDa β‐dystroglycan fragment. These data, therefore, support a cell density‐dependent γ‐secretase and furin mediated proteolysis of β‐dystroglycan, which could be notch stimulated, leading to nuclear targeting and subsequent degradation. 117: 2149–2157, 2016. © 2016 The Authors. *Journal of Cellular Biochemistry* Published by Wiley Periodicals, Inc.

Dystroglycan is an essential laminin binding receptor that exhibits aberrant posttranslational processing in most adenocarcinomas [Cross et al., 2008]. Furthermore, increased loss of dystroglycan is associated with poor disease outcome [Sgambato et al., 2007, 2010, 2003]. In adenocarcinoma, the extracellular α‐subunit of dystroglycan that interacts directly with laminin and other LG domain extracellular matrix proteins is frequently hypo‐glycosylated [Shimojo et al., 2011] due to transcriptional silencing of LARGE, a key glycosyltransferase required for the addition of O‐linked glycan moieties essential for the laminin G domain interaction (de Bernabe et al., 2009; Esser et al., 2013). The transmembrane β‐subunit, which interacts with α‐dystroglycan extracellularly and also connects to several different cytolinker proteins intracellularly [Moore and Winder, 2010], is also subject to altered N‐linked glycosylation [Singh et al., 2004; Mitchell et al., 2013]. Additional modifications to β‐dystroglycan, however, are phosphorylation on tyrosine [James et al., 2000; Sotgia et al., 2001], and specific proteolytic cleavage events [Losasso et al., 2000; Singh et al., 2004; Mitchell et al., 2013]. Tyrosine phosphorylation of β‐dystroglycan serves as a molecular switch to regulate the binding of different cellular binding partners [Moore and Winder, 2010], but is also a signal for the internalization of dystroglycan from the plasma membrane [Miller et al., 2012; Lipscomb et al., 2016], and may mediate some proteolytic events and nuclear translocation [Mathew et al., 2013; Mitchell et al., 2013]. β‐dystroglycan is subject to proteolysis at several key sites: matrix metalloproteinase‐mediated cleavage liberates the extracellular portion of β‐dystroglycan [Yamada et al., 2001; Zhong et al., 2006], the extracellular portion is not detectable as no antibody reagent to it exists, but the remaining 31 kDa transmembrane stub and cytoplasmic domain can be detected with antibodies to the carboxy‐terminus of the cytoplasmic domain. As yet unknown proteases generate smaller fragments corresponding to the cytoplasmic region of β‐dystroglycan [Losasso et al., 2000; Singh et al., 2004; Cross et al., 2008] most typically observed as a 26 kDa fragment, but occasionally a 17 kDa. Commonly used antibodies to β‐dystroglycan including those to the phosphorylated tyrosine residue, tyrosine 890, can detect all fragments retaining the c‐terminal 12 amino acids. Due to the presence of a nuclear localization signal in the cytoplasmic juxtamembrane region of β‐dystroglycan [Oppizzi et al., 2008], β‐dystroglycan and proteolytic fragments containing the nuclear localization signal can be targeted to the nucleus via an importin‐dependent pathway [Lara‐Chacon et al., 2010] where it can have effects on nuclear architecture [Martínez‐Vieyra et al., 2013]. Why the 43 kDa full length β‐dystroglycan, as well as the 31 kDa MMP‐cleaved and 26 kDa cytoplasmic fragment of β‐dystroglycan are all targeted to the nucleus is not clear. We have demonstrated previously in prostate cancer cell lines in vitro and in patient samples of prostate cancer, that a 26 kDa cytoplasmic fragment of dystroglycan is both phosphorylated on tyrosine and rapidly translocated to the nucleus [Mathew et al., 2013; Mitchell et al., 2013]. Furthermore, in LNCaP a prostate cancer cell, the nuclear translocation of a cytoplasmic fragment of β‐dystroglycan has been shown to be androgen‐dependent and leads to the transcription of some androgen‐regulated genes [Mathew et al., 2013]. We have, therefore, investigated the mechanisms leading to the proteolysis of β‐dystroglycan in order to understand the pathways leading to the nuclear targeting of dystroglycan in adenocarcinomas. Our findings suggest a cell density‐dependent γ‐secretase and furin mediated proteolysis of β‐dystroglycan, which may be stimulated by notch, leading to nuclear targeting and subsequent degradation.

## MATERIALS AND METHODS

Biochemical analysis of LNCaP cells, SDS–PAGE, and western blotting and maintenance of LNCaP cells was described previously [Mathew et al., 2013]. For nuclear fractionation, cells were rinsed in cold PBS and chilled on ice and harvested in the minimum volume of cold buffer 1 (0.32 M sucrose, 10 mM Tris–HCl pH 8.0, 3 mM calcium chloride, 2 mM magnesium acetate, 0.1 mM EDTA, 0.5% NP‐40, 1 mM DTT, 0.5 mM PMSF, and complete protease inhibitor mixture). Harvested cell lysates were homogenized using a cold dounce homogenizer on ice and centrifuged at 600*g* for 10 min at 4°C, pellet and supernatant were retained. The pellet was resuspended in buffer 1 and mixed with an equal volume of buffer 2 (2 M sucrose, 10 mM Tris–HCl pH 8.0, 5 mM magnesium acetate, 0.1 mM EDTA, 1 mM DTT, 0.5 mM PMSF, and complete protease inhibitor mixture). The resulting mixture was then carefully overlaid onto a 1.8 M sucrose cushion. Nuclei were then recovered by centrifugation through the sucrose cushion at 30,000*g* for 50 min. The nuclear pellet was resuspended directly in SDS–PAGE sample buffer and used as the nuclear fraction in immunoblotting experiments. The retained supernatant was centrifuged at 9300*g* for 10 min at 4°C and the resultant supernatant was used as the cytoplasmic fraction [Mathew et al., 2013].

Use of antibodies to non‐phosphorylated β‐dystroglycan (MANDAG2) [Pereboev et al., 2001], β‐dystroglycan phosphorylated on tyrosine 892 (1709) [Thompson et al., 2010], and fractionation purity and loading control antibodies α‐tubulin (T5168), lamin A/C (4C11) and GAPDH (GA1R) (Sigma, Gillingham, UK) have also been described as above [Mathew et al., 2013]. Western blots were developed using enhanced chemiluminescence, imaged using a Biorad ChemiDoc WRX+ and quantified using Image Lab software (Hemel Hempstead, UK).

LNCAP cells were treated at various concentrations and for various times as indicated in the figure legends, with one or more of the following: furin inhibitor 1 (Decanoyl‐RVKR‐CMK, Calbiochem, Watford, UK), γ‐secretase inhibitor DAPT (N‐[N‐(3,5‐Difluorophenacetyl‐l‐alanyl)[‐S‐phenylglycine t‐Butyl Ester, Calbiochem), proteasome inhibitor MG132 (Calbiochem), phorbol ester PDBu (phorbol 12,13‐dibutyrate, Sigma) and resveratrol (Enzo Life Sciences, Exeter, UK). Optimal incubation times for Furin Inhibitor 1 and DAPT were determined in preliminary experiments to be 24 h (data not shown). All compounds were dissolved in DMSO that was added to cells at a final concentration of no more than 1%, with an equivalent volume of DMSO used as a vehicle only control. Quantification of the levels of 26 kDa β‐dystroglycan fragment produced were expressed as a ratio to the amount of full‐length 43 kDa β‐dystroglycan present. Statistical analysis was carried out by Student's *t*‐test or ANOVA using Graphpad Prism 6 software (San Diego, CA).

## RESULTS

**Figure 1:**
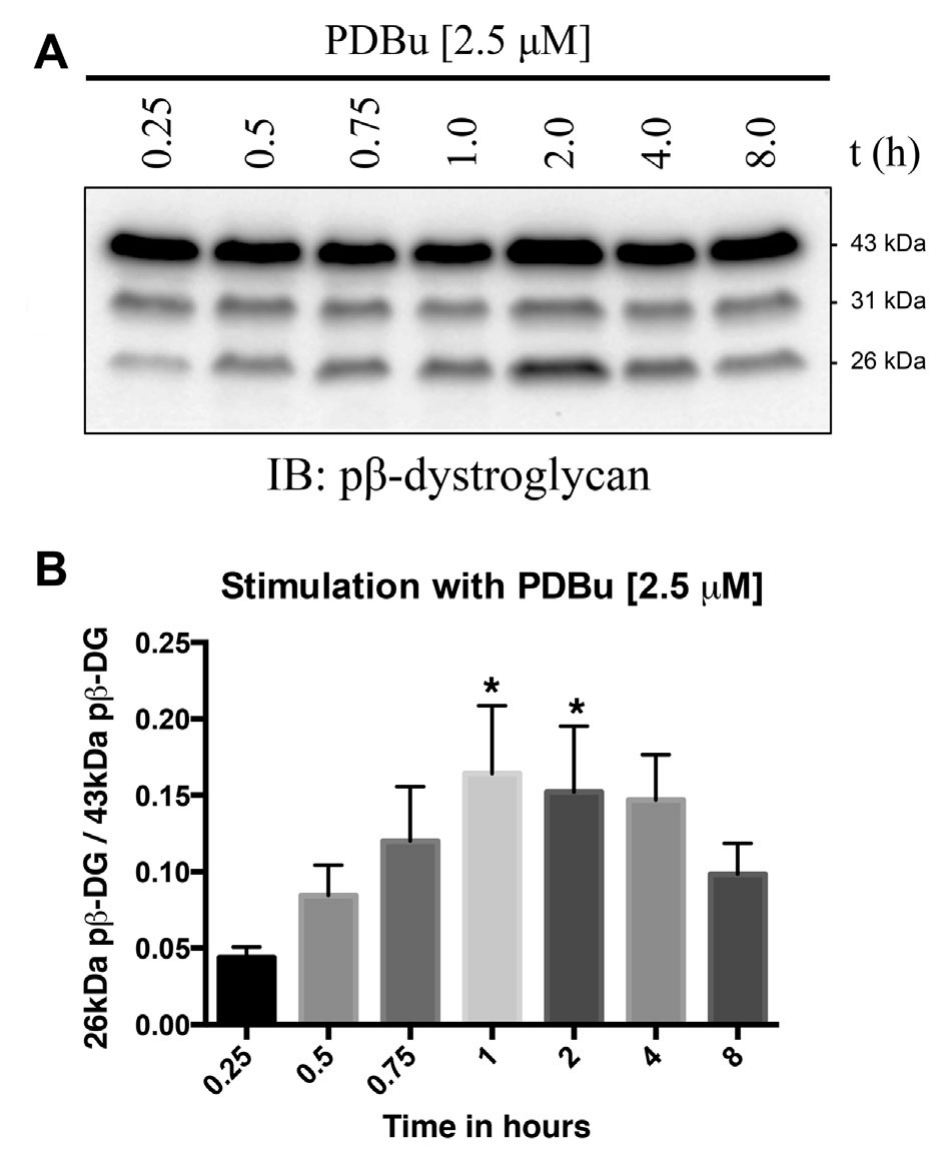
Phorbol ester‐induced proteolysis of β‐dystroglycan. Treatment of LNCaP cells with 2.5 μM phorbol 12,13‐dibutyrate (PDBu) for up to 8 h demonstrates a time‐dependent change in the proteolysis of phosphorylated β‐dystroglycan (A). The positions of the full‐length 43 kDa, ectodomain shed 31 kDa, and 26 kDa cytoplasmic fragments of β‐dystroglycan are indicated. PDBu leads to an increase in the levels of the 26 kDa fragment of β‐dystroglycan peaking at 1 h (B: mean ± sem of three independent experiments. *P < 0.05 Student's *t*‐test).

**Figure 2:**
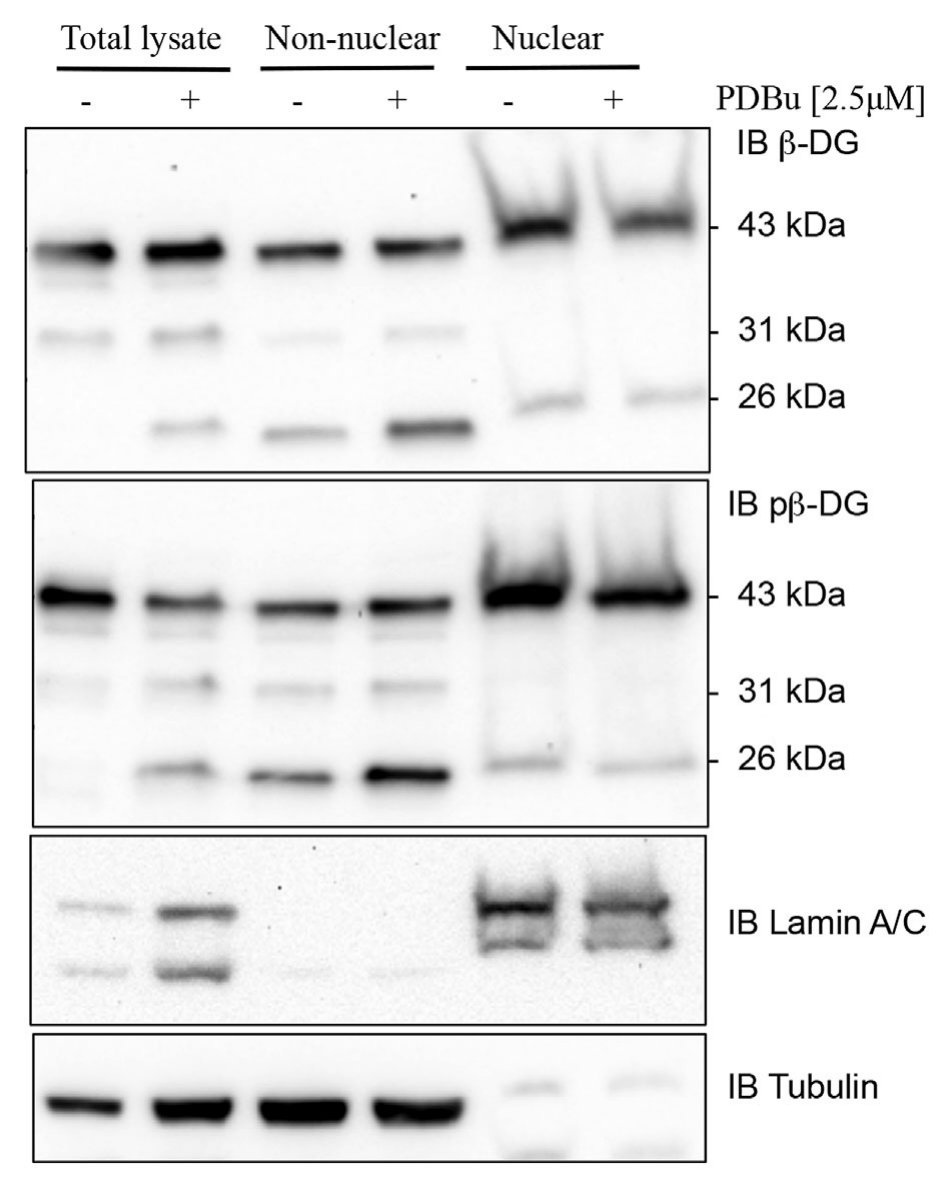
Dystroglycan levels in nuclear and non‐nuclear fractions. LNCaP cells were treated with 2.5 μM PDBu for 2 h and total cell lysates, and nuclear and non‐nuclear fractions were western blotted for non‐phosphorylated β‐dystroglycan (top panel) and phosphorylated β‐dystroglycan (middle panel). Lower two panels show lamin A/C nuclear fraction marker and loading control, and α‐tubulin non‐nuclear fraction marker and loading control. The nuclear fraction is clear of tubulin, and the cytoplasmic fraction is clear of lamin A/C. The relative positions of 43, 31, and 26 kDa fragments of β‐dystroglycan are indicated. A total of 43 kDa full length and the 26 kDa fragment but not 31 kDa β‐dystroglycan are present in the nucleus, although with no particular enrichment as a result of PDBu treatment. The slight mobility shift and smearing in the nuclear fractions is due to the ionic conditions and volume of sample loaded.

**Figure 3:**
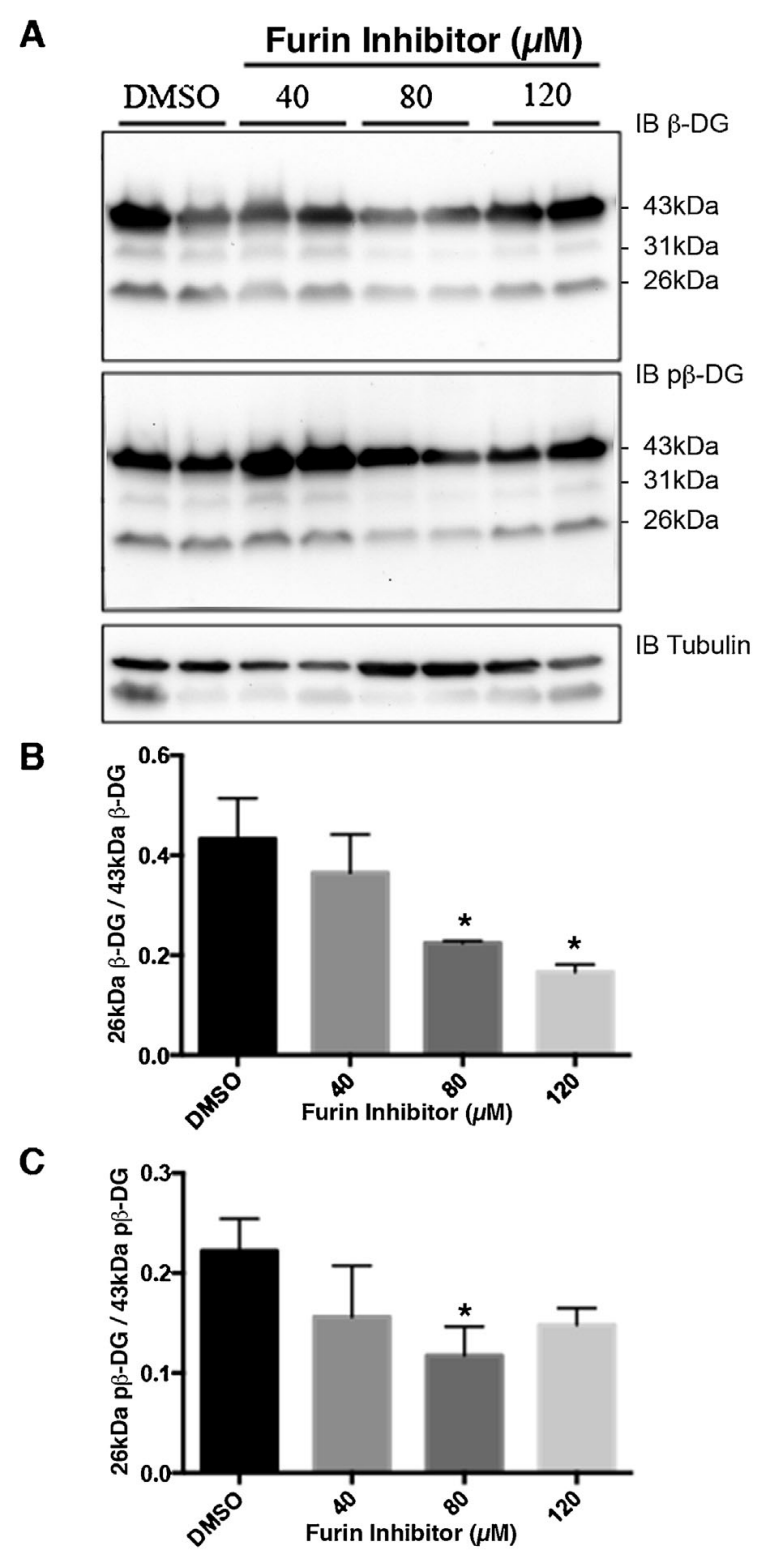
Furin inhibitor treatment of LNCaP cells reduces the levels of 26 kDa β‐dystroglycan. A: LNCaP cells were treated with the indicated levels of furin inhibitor 1 or DMSO control for 24 h. Total cell lysates were western blotted for β‐dystroglycan (β‐DG; top panel) or phosphorylated β‐dystroglycan (pβ‐DG; middle panel) with α‐tubulin as loading control. Blots were quantified and presented as the proportion of 26 kDa β‐dystroglycan/43 kDa β‐dystroglycan against furin inhibitor concentration (B) or for pβ‐dystroglycan (C). Data presented are the mean ± sem of four independent experiments. *P < 0.05 Student's *t*‐test.

**Figure 4:**
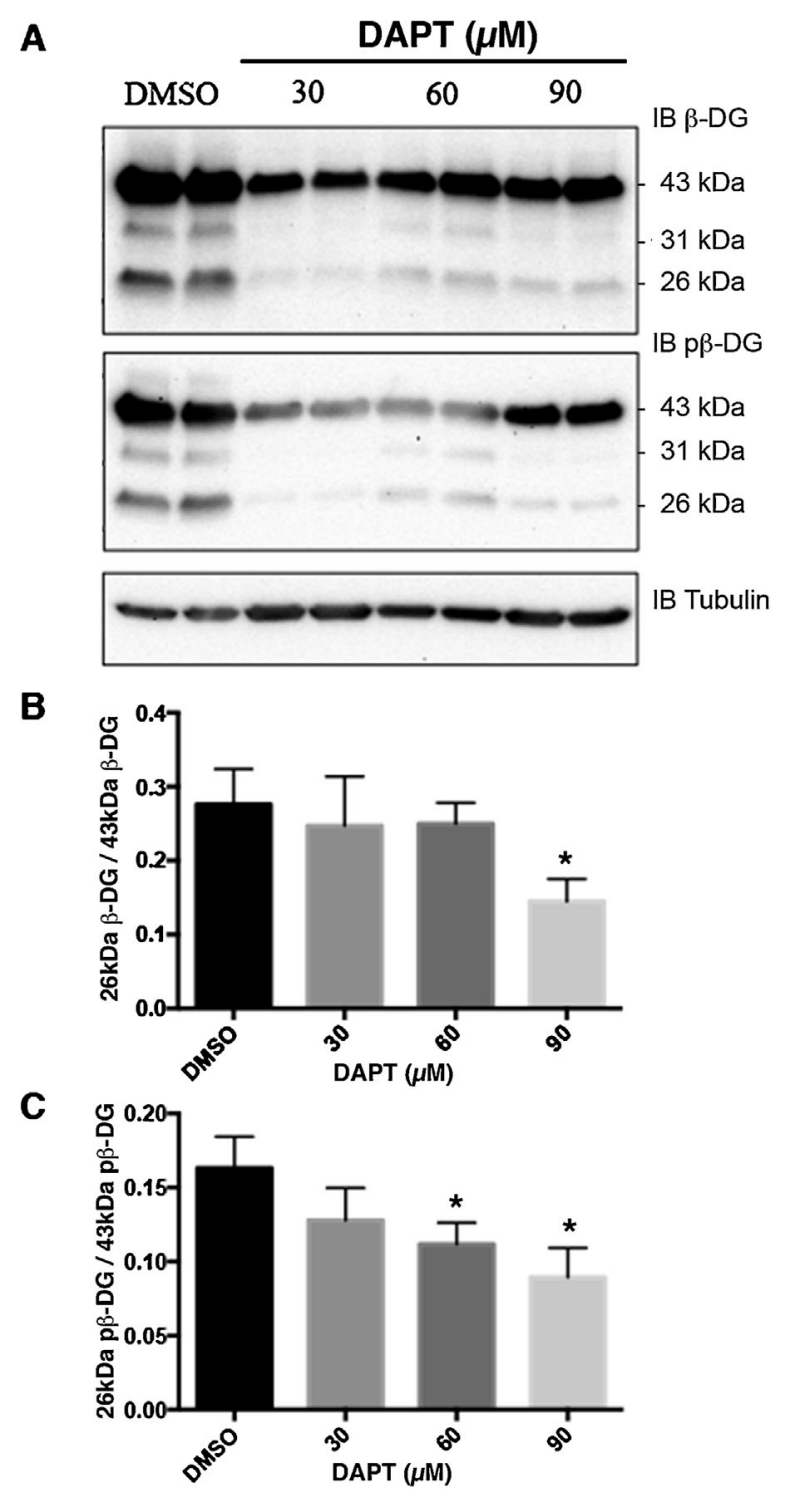
DAPT treatment of LNCaP cells reduces the levels of 26 kDa β‐dystroglycan. LNCaP cells were treated with the indicated levels of the γ‐secretase inhibitor DAPT or DMSO control for 24 h. A: Total cell lysates were western blotted for β‐dystroglycan (β‐DG; top panel) or phosphorylated β‐dystroglycan (pβ‐DG; middle panel) with α‐tubulin as loading control. Blots were quantified and presented as the proportion of 26 kDa β‐dystroglycan/43 kDa β‐dystroglycan against DAPT concentration (B), or for pβ‐dystroglycan (C). Data presented are the mean ± sem of six independent experiments. *P < 0.05 Student's *t*‐test.

β‐Dystroglycan undergoes proteolytic events dependent on cell culture conditions, such as cell density [Mitchell et al., 2013], in response to certain stimuli such as phorbol esters [Herzog et al., 2004] and in pathological conditions such as cancer and ischemia [Armstrong et al., 2003; Cross et al., 2008; Mitchell et al., 2013]. In silico analysis [Gould et al., 2010] of the dystroglycan sequence for potential proteolysis sites reveals the presence of potential furin cleavage sites in the juxtamembrane region of the cytoplasmic domain. Furin activity can be increased by protein kinase C (PKC) activation [Sundberg et al., 2004]. We have used PDBu previously and shown it to be a good stimulator of β‐dystroglycan phosphorylation and processing via PKC activation of Src in the formation of podosomes [Thompson et al., 2008]. Therefore, in order to stimulate PKC and through that increase furin activity, we treated LNCaP cells with the phorbol ester PDBu for various periods of time and examined the effect on dystroglycan. As can be seen in Figure 1, the relative abundance of the 26 kDa cytoplasmic region of dystroglycan increased significantly in a time dependent manner in response to PDBu treatment reaching a peak between 1 and 2 h of treatment and then declining. Extended phorbol ester treatment of cells is known to lead to down‐regulation of PKC activity, including in prostate cancer cells [Rusnak and Lazo, 1996]. Phorbol ester treatment can also lead to an activation of ADAM17 [Gooz, 2010]. However, the predicted cleavage site for ADAM17 is a valine with amino‐terminal alanine [Tucher et al., 2014], while such a consensus exists within the transmembrane region of β‐dystroglycan it is toward the extracellular face and would result in a fragment 2 kDa larger. No such fragments or doublets of fragments resulting from potential ADAM17 cleavage were observed on PDBu treatment (Fig. 1 and data not shown). β‐dystroglycan and in particular the 26 kDa fragment is known to translocate to the nucleus [Oppizzi et al., 2008; Lara‐Chacon et al., 2010; Martínez‐Vieyra et al., 2013; Mathew et al., 2013], furthermore, nuclear translocation is increased when β‐dystroglycan is phosphorylated on tyrosine [Mathew et al., 2013]. We, therefore, examined the effect of PDBu treatment on the generation of the 26 kDa fragment, and its phosphorylation status and nuclear localization. PDBu treatment of LNCaP cells increases the total cellular levels of the 26 kDa fragment of β‐dystroglycan, however, this does not appear to have a particularly robust effect on its either its phosphorylation status or its translocation to the nucleus. From the western blotting of the fractionation experiment shown in Figure 2, the largest concentration of 26 kDa β‐dystroglycan appears to be in response to PDBu stimulation and in the non‐nuclear fraction. Neither phosphorylation status nor the stimulation with PDBu appears to have any effect on the levels of 26 kDa β‐dystroglycan in the nuclear fraction (Fig. 2). Intra‐ or juxtamembrane cleavage of transmembrane proteins can be mediated by a number of distinct but related proteases. For example, activation of furin by PDBu and PKC can further lead to activation of secretases or MMPs. To unequivocally identify a role for furin in the cleavage of dystroglycan, we treated LNCaP cells with furin inhibitor 1 (Decanoyl‐RVKR‐CMK) at various concentrations and quantified the levels of the 26 kDa fragment. Furin inhibitor concentrations up to 120 μM had a dose dependent and significant effect on the amount of non‐phosphorylated 26 kDa dystroglycan, reducing levels by over 60%. The effects on the generation of the phosphorylated 26 kDa fragment were less pronounced, resulting in only a 35% reduction at 120 μM furin inhibitor (Fig. 3) suggesting the involvement of another type of proteolytic activity. As a previous proteomic screen had identified dystroglycan as a potential γ‐secretase substrate [Hemming et al., 2008] and to further delineate a possible proteolysis cascade, we treated LNCaP cells with the γ‐secretase inhibitor DAPT. Figure 4 shows an approximately 50% reduction in both un‐phosphorylated and tyrosine phosphorylated 26 kDa cytoplasmic region of β‐dystroglycan in response to γ‐secretase inhibition by DAPT, which are broadly similar in magnitude to the level of inhibition seen with the furin 1 inhibitor (Fig. 3). Administering PDBu and DAPT together did not reduce the amount of the 26 kDa fragment, while there was some reduction in the 31 kDa fragment (data not shown).

**Figure 5:**
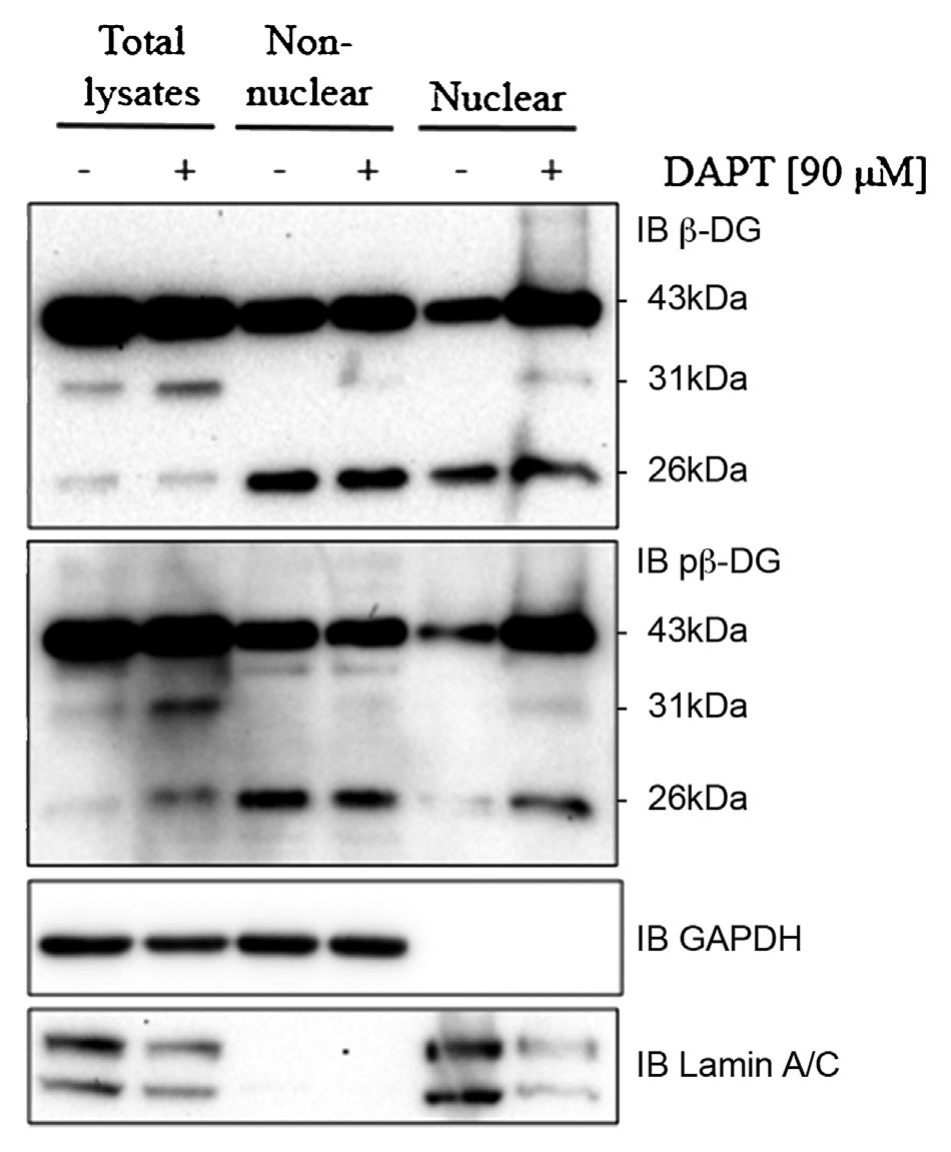
DAPT increases the levels of nuclear β‐dystroglycan. LNCaP cells were treated with 90 μM DAPT for 24 h and total cell lysates, and nuclear and non‐nuclear fractions were western blotted for non‐β‐dystroglycan (top panel) and phosphorylated β‐dystroglycan (middle panel). Lower two panels show lamin A/C nuclear fraction marker and loading control, and GAPDH non‐nuclear fraction marker and loading control. The relative position of β‐dystroglycan and fragments are indicated. Compared with the lamin A/C loading control all β‐dystroglycan species are increased in the nuclear fraction in LNCaP cells treated with DAPT.

It is well established that β‐dystroglycan and β‐dystroglycan fragments are trafficked to the inner nuclear membrane and the nucleoplasm, for example, see [Oppizzi et al., 2008; Martínez‐Vieyra et al., 2013]. Fractionation of control and DAPT treated LNCaP cells as shown in Figure 5 reveals no obvious effect on the native 43 or the 26 kDa β‐dystroglycan in the total lysates or non‐nuclear fraction. DAPT treatment does, however, result in a much higher level of the 31 kDa β‐dystroglycan fragment in the total cell lysate, both in its phosphorylated and non‐phosphorylated forms. Furthermore, in nuclear fractions, there is a very large increase in the levels 43 kDa β‐dystroglycan in both phosphorylated and non‐phosphorylated forms, and considering the protein loading relative to the lamin A/C loading control/purity marker, also a relative increase in the 31 kDa fragment as seen in the total lysates. There is a clear accumulation of the 31 kDa fragment in total lysates where γ‐secretase is inhibited compared with PDBu treated cells (compare Fig. 2). Paradoxically, there is also an increase in the phosphorylated form of the 26 kDa fragment in the nucleus. Proportional to the level of 43 kDa phosphorylated β‐dystroglycan, however, this still represents a relative decrease in 26 kDa levels.

**Figure 6:**
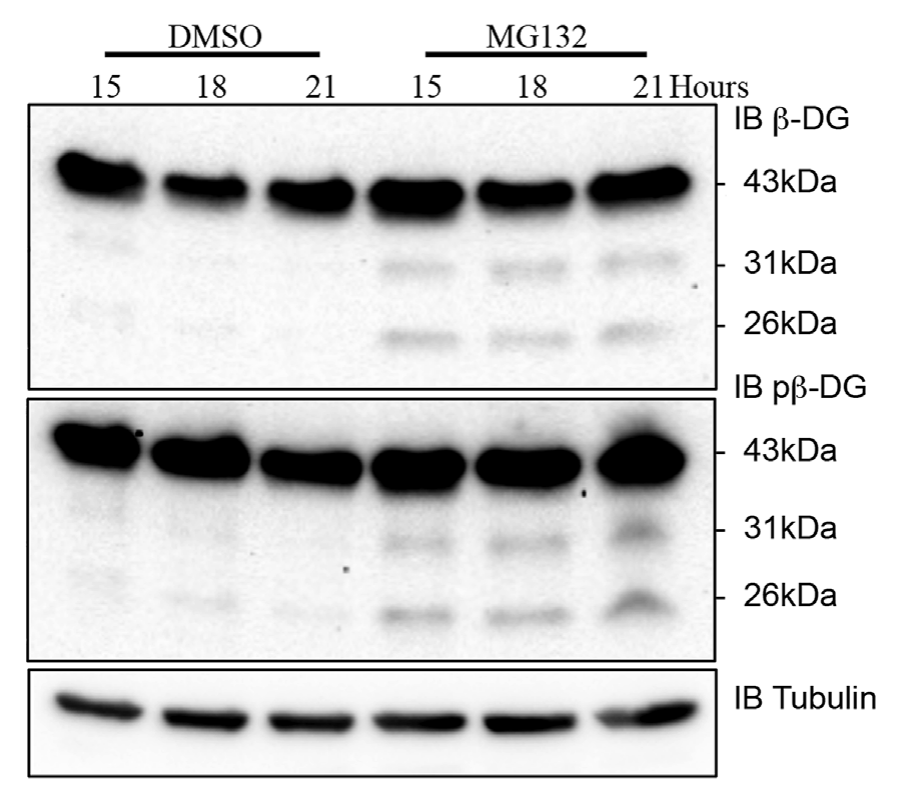
Proteasome inhibition increases the relative levels of β‐dystroglycan fragments. Compared with LNCaP cells treated with DMSO alone for up to 21 h, cell treated with 15 μM MG132 show a clear increase in both β‐dystroglycan or phosphorylated β‐dystroglycan. α‐tubulin is present as loading control.

**Figure 7:**
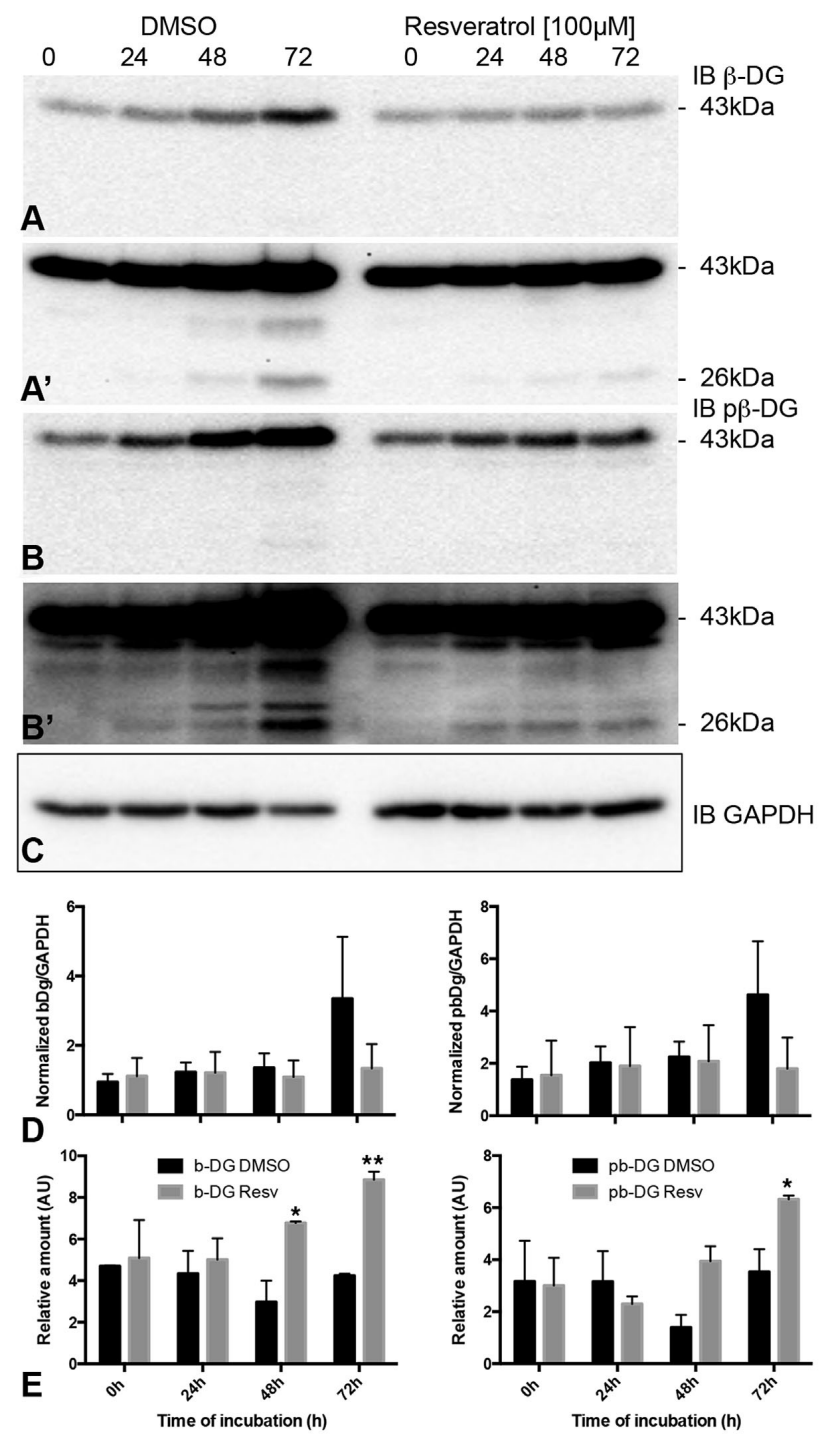
Resveratrol increases 26 kDa β‐dystroglycan levels. Treatment of sub‐confluent cultures of LNCaP cells with 100 μM resveratrol for up to 72 h resulted in an inhibition of the levels of β‐dystroglycan (A and A′) or phosphorylated β‐dystroglycan (B and B′) compared to DMSO only treated cells at 72 h as revealed by western blotting and quantification against a GAPDH loading control (C). Low exposure blots were used to quantify 43 kDa dystroglycan (A and B), and high exposure blots were used to quantify 26 kDa dystroglycan (A′ and B′). D: Quantification of three independent experiments against the GAPDH control revealed a halving of the levels of full length β‐dystroglycan at 72 h, whether phosphorylated or not. E: Quantification of the levels of 26 kDa β‐dystroglycan over the same time course revealed a resveratrol‐dependent doubling of the levels of 26 kDa β‐dystroglycan (phosphorylated and non‐phosphorylated) at 48 and 72 h of indication. Data are mean ± sem of three independent experiments in B and mean ± range of two independent experiments in C. *P < 0.05, **P < 0.05 two‐way ANOVA.

**Figure 8:**
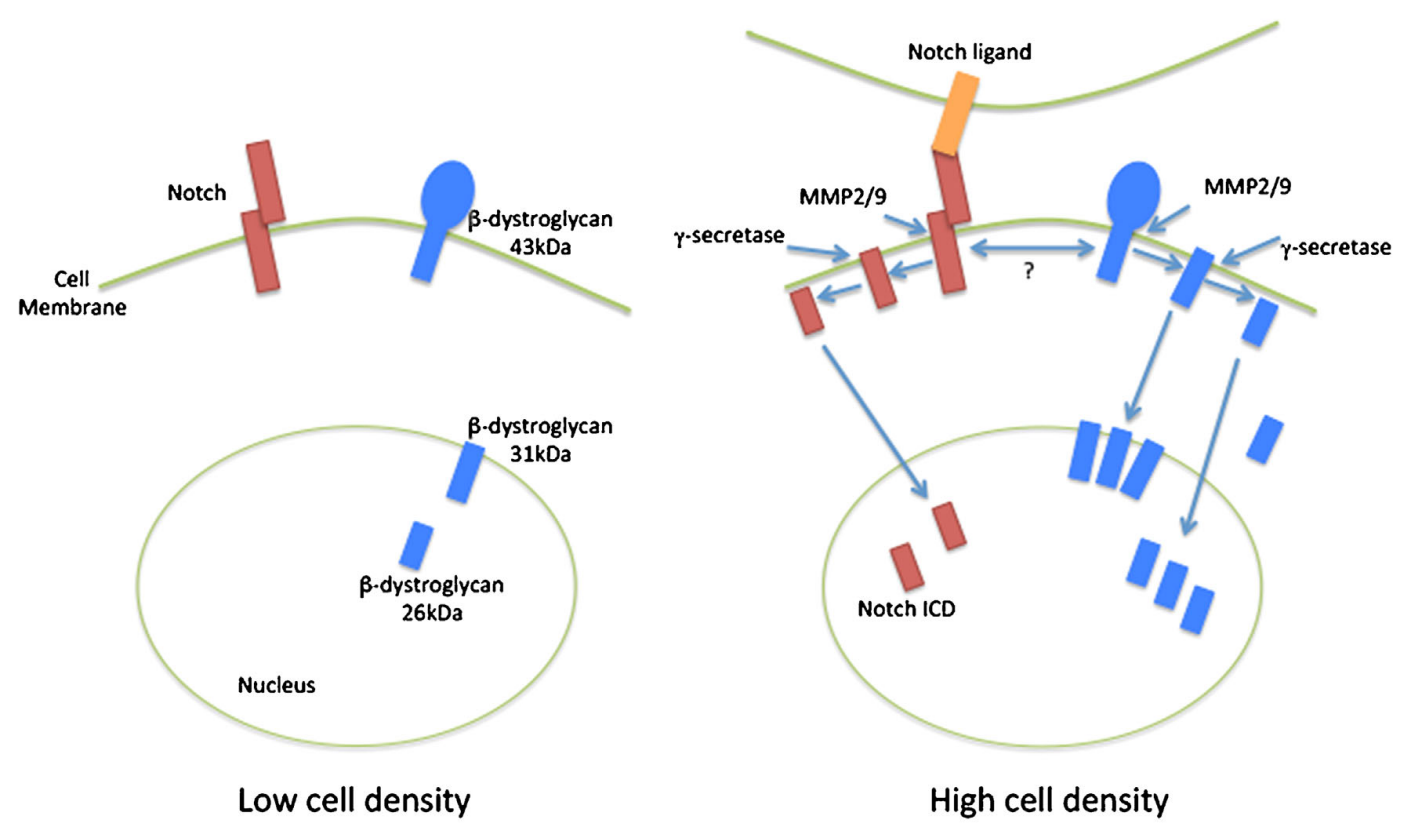
Schematic representation of a potential pathway leading to generation of β‐dystroglycan cleavage products and their translocation to the nucleus. In low cell density conditions, depicted on the left. Notch (in brown) is not engaged and there is a minimal amount of the 31 and 26 kDa β‐dystroglycan (in blue) cleavage products in the nucleus. However, as depicted on the right, on increased cell density, notch is activated leading to the shedding of the notch receptor and generation of the nuclear targeted notch ICD. MMP‐mediated cleavage of the β‐dystroglycan ectodomain is also stimulated and the levels of the 31 kDa fragment increase in the nucleus. Furthermore, similar to notch, an additional γ‐secretase mediated cleavage generates the 26 kDa cytoplasmic fragment of β‐dystroglycan which also accumulates in the nucleus.

These data could be interpreted as a cell density‐dependent plasma membrane cleavage event mediated by furin, trafficking of 31 kDa β‐dystroglycan to the nucleus where it then undergoes a further γ‐secretase mediated proteolytic cleavage event and is then degraded by the nuclear proteasome. Previous published findings revealed a cell density‐dependent increase in dystroglycan proteolysis and nuclear targeting [Mitchell et al., 2013]. Treatment of LNCaP cells with the proteasome inhibitor MG132 show that in fact both the 31 and 26 kDa fragments are preserved (Fig. 6), pointing to a mechanism whereby both can be degraded independently and not necessarily via a sequential process. High levels of MG132, typically at concentrations over 40 μM, can inhibit the action of secretases [Steinhilb et al., 2001]; however, MG132 was used at only 15 μM in this study. However, the increased levels of 31 kDa β‐dystroglycan observed in nuclear fractions treated with DAPT (Fig. 5) would suggest that the proportion of 31 kDa β‐dystroglycan that is trafficked to the nucleus does undergo a sequential series of proteolysis events. But what cellular signaling pathway is responsible for the apparent proteolysis and nuclear trafficking? The original observation came from LNCaP cells cultured at very high density [Mitchell et al., 2013], and one signaling pathway that is involved in sensing cell density and involves proteolysis and nuclear targeting is the Notch pathway [Kidd et al., 1998; Schroeter et al., 1998]. We, therefore, investigated if stimulating the Notch pathway pharmacologically but at low cell density, led to any changes in dystroglycan levels or the proteolysis of dystroglycan in LNCaP cells. Resveratrol treatment of subconfluent LNCaP cells for up to 72 h resulted in a proportional decrease in the total levels of both phosphorylated and non‐phosphorylated 43 kDa β‐dystroglycan (Fig. 7a and b). Conversely, quantification of the 26 kDa β‐dystroglycan bands from longer exposures of western blots from similar experiments, revealed a resveratrol stimulated time dependent and significant twofold increase in the phosphorylated form of 26 kDa β‐dystroglycan at 48 and 72 h of incubation (Fig. 7c). These data, therefore, support a cell density‐dependent γ‐secretase and furin mediated proteolysis of β‐dystroglycan, which may be stimulated by notch, leading to nuclear targeting and subsequent further degradation of β‐dystroglycan. These pathways are depicted in Figure 8.

## DISCUSSION

The post‐translational processing of dystroglycan is complex, not only is the α‐subunit extensively glycosylated, but the protein also undergoes co‐translational and post‐translational proteolytic cleavage events. The α‐ and β‐subunits of dystroglycan are themselves generated from the single pro‐peptide precursor by the actions of an endogenous SEA domain [Akhavan et al., 2008] and the α‐dystroglycan subunit in addition to the processing of its signal peptide undergoes a further furin‐dependent cleavage at the amino terminus to trim back the protein to the end of the mucin‐like domain [Singh et al., 2004]. Many publications investigating perturbations in dystroglycan function have focused on the appearance of lower molecular weight bands that are detectable with the commonly used antibodies that detect the c‐terminal 12 amino acids of β‐dystroglycan. The origins of these bands are somewhat enigmatic, and although the cleavage events have been frequently attributed to MMP activity, as well as furin and γ‐secretase [Singh et al., 2004; Agrawal et al., 2006; Zhong et al., 2006; Michaluk et al., 2007; Hemming et al., 2008; Bozzi et al., 2009; Mitchell et al., 2013], a physiologically relevant stimulus–effect relationship has not been established. Furthermore, the site of cleavage may determine the fate and function of the remaining cellular β‐dystroglycan fragment. Hemming et al. [2008] in their analysis of γ‐secretase cleavage sites in cell adhesion receptors demonstrated that in order to visualize the products of γ‐secretase mediated cleavage of β‐dystroglycan, one had to inhibit the proteasome with epoxomicin. Although this cleavage was artificial, in that the substrate was an overexpressed fusion protein with cytoplasmic Flag‐tag at the carboxy terminus, the resulting fragments were clearly subjected to fairly rapid proteasomal degradation. Indeed, the 26 kDa fragment was visible only in epoxomicin treated cells [Hemming et al., 2008]. In this study phorbol ester or resveratrol treatment of low density LNCaP cells revealed an increase in the amount of the 26 kDa fragment, which was efficiently targeted to the nucleus. A potential notch‐dependent pathway of dystroglycan proteolysis and nuclear targeting with subsequent effects on transcriptional regulation would have parallels with the nuclear targeting of intracellular domains from other receptors, such as notch itself [Kidd et al., 1998; Schroeter et al., 1998], or CD44 [Okamoto et al., 2001; Lammich et al., 2002] for example. Despite a role for nuclear dystroglycan in regulating the transcriptional activity of a number of genes [Mathew et al., 2013], dystroglycan itself was not upregulated, providing a possible explanation for the drop in dystroglycan levels upon resveratrol treatment, as seen in Figure 7. Nonetheless, cell density does increase the levels of full length and cytoplasmic cleaved dystroglycan and increases the levels of cytoplasmic dystroglycan targeted to the nucleus [Mathew et al., 2013; Mitchell et al., 2013]. One of the genes significantly upregulated by the targeting of the 26 kDa dystroglycan fragment to the nucleus is the androgen responsive gene ETV1 [Mathew et al., 2013]. Interestingly, an analysis of the mouse dystroglycan promoter regions revealed a putative androgen response element [Rettino et al., 2009] raising the possibility of a more complex interdependent transcriptional regulatory network involving both androgens, and potentially, notch‐dependent nuclear targeting of dystroglycan. As we have demonstrated previously [Mitchell et al., 2013], loss of dystroglycan upon increased cell density was permissive for LNCaP three‐dimensional and anchorage‐independent growth. From those studies and those presented here, we can now propose that the mechanism governing those changes may be mediated by notch. Notch mediated changes to β‐dystroglycan in concert with the depletion of α‐dystroglycan laminin binding by hypo‐glycosylation [Bao et al., 2009; de Bernabe et al., 2009; Shimojo et al., 2011; Esser et al., 2013] is likely to contribute significantly to the tomourigenic potential of prostate cancer. Moreover, this may be a general mechanism that is likely to be relevant to the majority of adenocarcinomas where dystroglycan function is also abrogated [Sgambato et al., 2003; Cross et al., 2008].

## References

[jcb25537-bib-0001] Agrawal S , Anderson P , Durbeej M , van Rooijen N , Ivars F , Opdenakker G , Sorokin LM . 2006 Dystroglycan is selectively cleaved at the parenchymal basement membrane at sites of leukocyte extravasation in experimental autoimmune encephalomyelitis. J Exp Med 203:1007–1019. 16585265 10.1084/jem.20051342 PMC2118280

[jcb25537-bib-0002] Akhavan A , Crivelli SN , Singh M , Lingappa VR , Muschler JL . 2008 SEA domain proteolysis determines the functional composition of dystroglycan. FASEB J 22:612–621. 17905726 10.1096/fj.07-8354com

[jcb25537-bib-0003] Armstrong SC , Latham CA , Ganote CE . 2003 An ischemic b‐dystroglycan degradation product: Correlation with irreversible injury in adult rabbit cardiomyocytes. Mol Cell Biochem 242:71–79. 12619868

[jcb25537-bib-0004] Bao X , Kobayashi M , Hatakeyama S , Angata K , Gullberg D , Nakayama J , Fukuda MN , Fukuda M . 2009 Tumor suppressor function of laminin‐binding alpha‐dystroglycan requires a distinct beta3‐N‐acetylglucosaminyltransferase. Proc Natl Acad Sci USA 106:12109–12114. 19587235 10.1073/pnas.0904515106 PMC2707272

[jcb25537-bib-0005] Bozzi M , Inzitari R , Sbardell D , Monaco S , Pavoni E , Gioia M , Marini S , Morlacchi S , Sciandra F , Castagnola M , Giardina B , Brancaccio A , Coletta M . 2009 Enzymatic processing of β‐dystroglycan recombinant ectodomain by MMP‐9: Identification of the main cleavage site. IUBMB Life 61:1143–1152. 19946898 10.1002/iub.273

[jcb25537-bib-0006] Cross S , Lippitt J , Mitchell A , Hollingsbury F , Balasubramanian S , Reed M , Eaton C , Catto J , Hamdy F , Winder S . 2008 The expression of β‐dystroglycan is reduced or absent in the majority of human carcinomas. Histopathology 53:561–566. 18983465 10.1111/j.1365-2559.2008.03157.x

[jcb25537-bib-0007] de Bernabe DB‐V , Inamori K‐I , Yoshida‐Moriguchi T , Weydert CJ , Harper HA , Willer T , Henry MD , Campbell KP . 2009 Loss of a‐dystroglycan laminin binding in epithelium‐derived cancers is caused by silencing of LARGE. J Biol Chem 284:11279–11284. 19244252 10.1074/jbc.C900007200 PMC2670132

[jcb25537-bib-0008] Esser AK , Miller MR , Huang Q , Meier MM , Beltran‐Valero de Bernabe D , Stipp CS , Campbell KP , Lynch CF , Smith BJ , Cohen MB , Henry MD . 2013 Loss of LARGE2 disrupts functional glycosylation of alpha‐dystroglycan in prostate cancer. J Biol Chem 288:2132–2142. 23223448 10.1074/jbc.M112.432807 PMC3554886

[jcb25537-bib-0009] Gooz M . 2010 ADAM‐17: The enzyme that does it all. Crit Rev Biochem Mol Biol 45:146–169. 20184396 10.3109/10409231003628015 PMC2841225

[jcb25537-bib-0010] Gould CM , Diella F , Via A , Puntervoll P , Gemund C , Chabanis‐Davidson S , Michael S , Sayadi A , Bryne JC , Chica C , Seiler M , Davey NE , Haslam N , Weatheritt RJ , Budd A , Hughes T , Pas J , Rychlewski L , Trave G , Aasland R , Helmer‐Citterich M , Linding R , Gibson TJ . 2010 ELM: The status of the 2010 eukaryotic linear motif resource. Nucleic Acids Res 38:D167–D180. 19920119 10.1093/nar/gkp1016 PMC2808914

[jcb25537-bib-0011] Hemming ML , Elias JE , Gygi SP , Selkoe DJ . 2008 Proteomic profiling of gamma‐secretase substrates and mapping of substrate requirements. PLoS Biol 6:e257. 18942891 10.1371/journal.pbio.0060257 PMC2570425

[jcb25537-bib-0012] Herzog C , Has C , Franzke CW , Echtermeyer FG , Schlotzer‐Schrehardt U , Kroger S , Gustafsson E , Fassler R , Bruckner‐Tuderman L . 2004 Dystroglycan in skin and cutaneous cells: Beta‐subunit is shed from the cell surface. J Invest Dermatol 122:1372–1380. 15175026 10.1111/j.0022-202X.2004.22605.x

[jcb25537-bib-0013] James M , Nuttall A , Ilsley JL , Ottersbach K , Tinsley JN , Sudol M , Winder SJ . 2000 Adhesion‐dependent tyrosine phosphorylation of b‐dystroglycan regulates its interaction with utrophin. J Cell Sci 113:1717–1726. 10769203 10.1242/jcs.113.10.1717

[jcb25537-bib-0014] Kidd S , Lieber T , Young MW . 1998 Ligand‐induced cleavage and regulation of nuclear entry of notch in *Drosophila melanogaster* embryos. Genes Dev 12:3728–3740. 9851979 10.1101/gad.12.23.3728 PMC317253

[jcb25537-bib-0015] Lammich S , Okochi M , Takeda M , Kaether C , Capell A , Zimmer AK , Edbauer D , Walter J , Steiner H , Haass C . 2002 Presenilin‐dependent intramembrane proteolysis of CD44 leads to the liberation of its intracellular domain and the secretion of an Abeta‐like peptide. J Biol Chem 277:44754–44759. 12223485 10.1074/jbc.M206872200

[jcb25537-bib-0016] Lara‐Chacon B , de Leon MB , Leocadio D , Gomez P , Fuentes‐Mera L , Martinez‐Vieyra I , Ortega A , Jans DA , Cisneros B . 2010 Characterization of an Importin alpha/beta‐recognized nuclear localization signal in beta‐dystroglycan. J Cell Biochem 110:706–717. 20512930 10.1002/jcb.22581

[jcb25537-bib-0017] Lipscomb L , Piggott RW , Emmerson T , Winder SJ . 2016 Dasatinib as a treatment for Duchenne muscular dystrophy. Hum Mol Genet 25:266–274. 26604135 10.1093/hmg/ddv469 PMC4706114

[jcb25537-bib-0018] Losasso C , Di Tommaso F , Sgambato A , Ardito R , Cittadini A , Giardina B , Petrucci TC , Brancaccio A . 2000 Anomalous dystroglycan in carcinoma cell lines. FEBS Lett 484:194–198. 11078877 10.1016/s0014-5793(00)02157-8

[jcb25537-bib-0019] Martínez‐Vieyra IA , Vásquez‐Limeta A , González‐Ramírez R , Morales‐Lázaro SL , Mondragón M , Mondragón R , Ortega A , Winder SJ , Cisneros B . 2013 A role for b‐dystroglycan in the organization and structure of the nucleus in myoblasts. Biochim Biophys Acta Mol Cell Res 1833:698–711. 10.1016/j.bbamcr.2012.11.019 23220011

[jcb25537-bib-0020] Mathew G , Mitchell A , Down JM , Jacobs LA , Hamdy FC , Eaton C , Rosario DJ , Cross SS , Winder SJ . 2013 Nuclear targeting of dystroglycan promotes the expression of androgen regulated transcription factors in prostate cancer. Sci Rep 3:2792. 24077328 10.1038/srep02792 PMC3786294

[jcb25537-bib-0021] Michaluk P , Kolodziej L , Mioduszewska B , Wilczynski GM , Dzwonek J , Jaworski J , Gorecki DC , Ottersen OP , Kaczmarek L . 2007 Beta‐dystroglycan as a target for MMP‐9, in response to enhanced neuronal activity. J Biol Chem 282:16036–16041. 17426029 10.1074/jbc.M700641200

[jcb25537-bib-0022] Miller G , Moore CJ , Terry R , Riviere TL , Mitchell A , Piggott R , Dear TN , Wells DJ , Winder SJ . 2012 Preventing phosphorylation of dystroglycan ameliorates the dystrophic phenotype in mdx mouse. Hum Mol Gen 21:4508–4520. 22810924 10.1093/hmg/dds293 PMC5886373

[jcb25537-bib-0023] Mitchell A , Mathew G , Jiang T , Hamdy F , Cross S , Eaton C , Winder S . 2013 Dystroglycan function is a novel determinant of tumour growth and behavior in prostate cancer. Prostate 73:398–408. 22996647 10.1002/pros.22581

[jcb25537-bib-0024] Moore CJ , Winder SJ . 2010 Dystroglycan versatility in cell adhesion: A tale of multiple motifs. Cell Commun Signal 8:3. 20163697 10.1186/1478-811X-8-3 PMC2834674

[jcb25537-bib-0025] Okamoto I , Kawano Y , Murakami D , Sasayama T , Araki N , Miki T , Wong AJ , Saya H . 2001 Proteolytic release of CD44 intracellular domain and its role in the CD44 signaling pathway. J Cell Biol 155:755–762. 11714729 10.1083/jcb.200108159 PMC2150876

[jcb25537-bib-0026] Oppizzi ML , Akhavan A , Singh M , Fata JE , Muschler JL . 2008 Nuclear translocation of b‐dystroglycan reveals a distinctive trafficking pattern of autoproteolyzed mucins. Traffic 9:2063–2072. 18764929 10.1111/j.1600-0854.2008.00822.x PMC2950207

[jcb25537-bib-0027] Pereboev AV , Ahmed N , thi Man N , Morris GE . 2001 Epitopes in the interacting regions of beta‐dystroglycan (PPxY motif) and dystrophin (WW domain). Biochim Biophys Acta 1527:54–60. 11420143 10.1016/s0304-4165(01)00147-7

[jcb25537-bib-0028] Rettino A , Rafanelli F , Genovese G , Goracci M , Cifarelli RA , Cittadini A , Sgambato A . 2009 Identification of Sp1 and GC‐boxes as transcriptional regulators of mouse Dag1 gene promoter. Am J Physiol Cell Physiol 297:C1113–C1123. 19657058 10.1152/ajpcell.00189.2009

[jcb25537-bib-0029] Rusnak JM , Lazo JS . 1996 Downregulation of protein kinase C suppresses induction of apoptosis in human prostatic carcinoma cells. Exp Cell Res 224:189–199. 8612685 10.1006/excr.1996.0127

[jcb25537-bib-0030] Schroeter EH , Kisslinger JA , Kopan R . 1998 Notch‐1 signalling requires ligand‐induced proteolytic release of intracellular domain. Nature 393:382–386. 9620803 10.1038/30756

[jcb25537-bib-0031] Sgambato A , Camerini A , Amoroso D , Genovese G , De Luca F , Cecchi M , Migaldi M , Rettino A , Valsuani C , Tartarelli G , Donati S , Siclari O , Rossi G , Cittadini A . 2007 Expression of dystroglycan correlates with tumor grade and predicts survival in renal cell carcinoma. Cancer Biol Ther 6:1840–1846. 18087214 10.4161/cbt.6.12.4983

[jcb25537-bib-0032] Sgambato A , Camerini A , Genovese G , De Luca F , Viacava P , Migaldi M , Boninsegna A , Cecchi M , Sepich CA , Rossi G , Arena V , Cittadini A , Amoroso D . 2010 Loss of nuclear p27kip1 and α‐dystroglycan is a frequent event and is a strong predictor of poor outcome in renal cell carcinoma. Cancer Science 101:2080–2086. 20626751 10.1111/j.1349-7006.2010.01644.x PMC11159623

[jcb25537-bib-0033] Sgambato A , Migaldi M , Montanari M , Camerini A , Brancaccio A , Rossi G , Cangiano R , Losasso C , Capelli G , Trentini GP , Cittadini A . 2003 Dystroglycan expression is frequently reduced in human breast and colon cancers and is associated with tumor progression. Am J Pathol 162:849–860. 12598319 10.1016/S0002-9440(10)63881-3 PMC1868099

[jcb25537-bib-0034] Shimojo H , Kobayashi M , Kamigaito T , Shimojo Y , Fukuda M , Nakayama J . 2011 Reduced glycosylation of α‐dystroglycans on carcinoma cells contributes to formation of highly infiltrative histological patterns in prostate cancer. Prostate 71:1151–1157. 21656825 10.1002/pros.21330 PMC3174275

[jcb25537-bib-0035] Singh J , Itahana Y , Knight‐Krajewski S , Kanagawa M , Campbell KP , Bissell MJ , Muschler J . 2004 Proteolytic enzymes and altered glycosylation modulate dystroglycan function in carcinoma cells. Cancer Res 64:6152–6159. 15342399 10.1158/0008-5472.CAN-04-1638

[jcb25537-bib-0036] Sotgia F , Lee H , Bedford M , Petrucci TC , Sudol M , Lisanti MP . 2001 Tyrosine phosphorylation of b‐dystroglycan at its WW domain binding motif, PPxY, recruits SH2 domain containing proteins. Biochemistry 40:14585–14592. 11724572 10.1021/bi011247r

[jcb25537-bib-0037] Steinhilb ML , Turner RS , Gaut JR . 2001 The protease inhibitor, MG132, blocks maturation of the amyloid precursor protein Swedish mutant preventing cleavage by β‐secretase. J Biol Chem 276:4476–4484. 11084038 10.1074/jbc.M008793200

[jcb25537-bib-0038] Sundberg C , Thodeti CK , Kveiborg M , Larsson C , Parker P , Albrechtsen R , Wewer UM . 2004 Regulation of ADAM12 cell‐surface expression by protein kinase C ε. J Biol Chem 279:51601–51611. 15364951 10.1074/jbc.M403753200

[jcb25537-bib-0039] Thompson O , Kleino I , Crimaldi L , Gimona M , Saksela K , Winder SJ . 2008 Dystroglycan, Tks5 and Src mediated assembly of podosomes in myoblasts. PLoS ONE 3:e3638. 18982058 10.1371/journal.pone.0003638 PMC2572840

[jcb25537-bib-0040] Thompson O , Moore CJ , Hussain S‐A , Kleino I , Peckham M , Hohenester E , Ayscough KR , Saksela K , Winder SJ . 2010 Modulation of cell spreading and cell‐substrate adhesion dynamics by dystroglycan. J Cell Sci 123:118–127. 20016072 10.1242/jcs.047902 PMC2794713

[jcb25537-bib-0041] Tucher J , Linke D , Koudelka T , Cassidy L , Tredup C , Wichert R , Pietrzik C , Becker‐Pauly C , Tholey A . 2014 LC‐MS based cleavage site profiling of the proteases ADAM10 and ADAM17 using proteome‐derived peptide libraries. J Proteome Res 13:2205–2214. 24635658 10.1021/pr401135u

[jcb25537-bib-0042] Yamada H , Saito F , Fukuta‐Ohi H , Zhong D , Hase A , Arai K , Okuyama A , Maekawa R , Shimizu T , Matsumura K . 2001 Processing of b‐dystroglycan by matrix metalloproteinase disrupts the link between the extracellular matrix and cell membrane via the dystroglycan complex. Hum Mol Genet 10:1563–1569. 11468274 10.1093/hmg/10.15.1563

[jcb25537-bib-0043] Zhong D , Saito F , Saito Y , Nakamura A , Shimizu T , Matsumura K . 2006 Characterization of the protease activity that cleaves the extracellular domain of beta‐dystroglycan. Biochem Biophys Res Commun 345:867–871. 16701552 10.1016/j.bbrc.2006.05.004

